# Review: questionnaires as measures for low energy availability (LEA) and relative energy deficiency in sport (RED-S) in athletes

**DOI:** 10.1186/s40337-021-00396-7

**Published:** 2021-03-31

**Authors:** Alexiaa Sim, Stephen F. Burns

**Affiliations:** Physical Education and Sports Science, National Institute of Education, Nanyang Technological University, 1 Nanyang Walk, Singapore, 637616 Singapore

**Keywords:** Low energy availability, Energy deficiency, Relative energy deficiency in sport, Female athlete triad syndrome, Feeding and eating disorders, mental disorders

## Abstract

**Background:**

A sustained mismatch between energy intake and exercise energy expenditure (EEE) can lead to Low Energy Availability (LEA), health and performance impairments characteristic of Relative Energy Deficiency in Sport (RED-S). Questionnaires can conveniently identify symptoms and/or LEA/ RED-S risk factors. This study aimed to systematically identify, and critique questionnaires used or developed to measure LEA/ RED-S risk in athletic populations.

**Methods:**

A systematic search was conducted using PubMed database. Full text articles were included if: (i) the questionnaire(s) in the study identified LEA and/or RED-S risk; (ii) studies developed questionnaires to identify LEA and/or RED-S risk; (iii) participants belonged to athletic population(s); and (iv) in English.

**Results:**

Thirty-three articles met the inclusion criteria and were reviewed, 13 questionnaires were identified. Eight questionnaires had undergone validation procedures, and three questionnaires included questions related to EEE. The most widely used validated questionnaires were Low Energy Availability in Females Questionnaire (LEAF-Q) (48% articles) and Eating Disorder Examination Questionnaire (EDE-Q) (12% articles). The LEAF-Q determines LEA risk from symptoms but cannot be used in males as nearly half of the items (*n* = 12) relate to menstrual function. The EDE-Q serves as a surrogate marker of LEA risk in both sexes, as it measures a major risk factor of LEA, disordered eating. Better validation is needed for many questionnaires and more are needed to address LEA/RED-S risk in male athletes.

**Conclusion:**

These questionnaires may be effective in identifying intentional energy restriction but less valuable in identifying inadvertently failure to increase energy intake with increased EEE.

## Plain English summary

Participating in physical activities and exercise can bring about numerous health benefits, especially when the body is properly fuelled with sufficient energy. However, failure to consume enough energy to provide for exercise and daily living can lead to a state of Low Energy Availability (LEA). This can be caused by disordered eating behaviours and/or excessive exercising. LEA can lead to the manifestation of Relative Energy Deficiency in Sport (RED-S), a condition that can result in irreversible health and performance impairments. RED-S can impact both females and males. Hence it is important to prevent LEA/ RED-S through regular screening of at-risk populations (e.g. athletes). Current methods of LEA/ RED-S risk screening require extensive resources which are difficult to access, other than in clinical settings. This review aimed to identify and critique questionnaires that have identified or addressed LEA/ RED-S risk. Questionnaires can be a useful, convenient, and relatively simple method for screening or early detection of LEA/ RED-S. However, they should not serve as diagnostic tools. Should questionnaires indicate any LEA/ RED-S risks, a clinical follow-up is necessary to prevent escalation of the condition, to safeguard athletes’ health and performance.

## Introduction

**Fig. 1:**
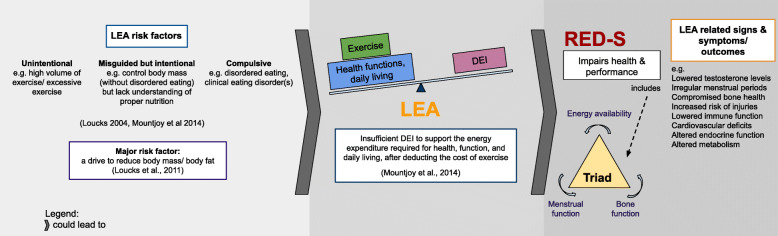
Unintentional, misguided but intentional, and compulsive behaviors are risk factors for low energy availability (LEA). These risk factors can result in a decrease in Dietary Energy Intake (DEI) and/ or increase in exercise energy expenditure (EEE). Overtime, these lead to Relative Energy Deficiency in Sport (RED-S), with concomitant health and performance consequences. These can present as signs, symptoms and outcomes in both male (e.g. lowered testosterone levels) and female (e.g. irregular menstrual cycle) athletes. RED-S encompasses the earlier identified condition Female Athlete Triad (Triad)

Low Energy Availability (LEA) occurs when an individual fails to consume sufficient energy to cover the exercise energy expenditure (EEE), and maintain basic physiological functions [1]. LEA is related to inadequate dietary energy intake (DEI) and/or high EEE [2], and it’s occurrence is associated with risk factors such as compulsive disordered eating, mismanaged and misinformed eating and compulsive exercising behaviours [2]. Thus, LEA can occur intentionally in a compulsive manner in pursuit of a specific body size or shape. However, it can also arise from mismanaged rational efforts to achieve a certain body size or fatness for athletic competitions which may not include disordered eating behaviours, or alternatively from unintentional dietary inadequacy such as the failure to increase DEI to compensate for an increase in the EEE [3]. If persistent, LEA results in the physiological disruption of the body including menstrual function, bone health, metabolism, immunity and cardiovascular health (Fig. 1).

LEA is the etiological factor underpinning the condition, Relative Energy Deficiency in Sport (RED-S) [4]. The International Olympic Committee (IOC) established RED-S, which supersedes the Female Athlete Triad (Triad) [4], in part because multiple body systems beyond menstrual function and bone are severely impacted by LEA along with performance. Moreover, LEA affects both male and female athletes [4], although the severity of abnormalities associated with LEA and the development of RED-S may differ between sexes. These abnormalities include compromised bone health, metabolic abnormalities, menstrual dysfunctions, decreased immune function, cardiovascular deficits, and altered endocrine function – these may be detrimental to athletes' health and potentially irreversible in the long-term [4].

Since the release of the IOC consensus papers in 2014 and 2018 [4, 5], research on RED-S and LEA has increased and certainly the prevalence of LEA is of concern. Recent studies have shown that 45% of female recreational exercises were found to be at risk of LEA [6], while another study found a high LEA prevalence in both male and female elite young athletes (males, 56%; females, 51%) [7]. However, reliable screening tools for at-risk athletic populations remain equivocal despite the importance of early detection to prevent long-term health implications. The RED-S Clinical Assessment Tool (RED-S CAT) [8] is the preferred clinical tool and employs a ‘Red Light - Yellow Light - Green Light’ risk assessment. However, better validation is needed for this as there appears to be some consensus with existing tools on who is at risk, but less agreement on the extent of the risk and case decisions [9, 10]. Part of this is because there is no comprehensive agreement or gold standard list of risk factors and symptoms for RED-S, probably because it affects so many body systems. For example, the REDS-CAT highlights that screening and diagnosis of RED-S is challenging and symptomatology subtle. Symptoms involve a wide-range of physiological (e.g. low body fat, reduced bone mineral density, absence of menstrual cycle, electrocardiogram abnormalities, and recurrent illnesses and injuries), psychological (depression, anxiety, mood changes and measures of disordered eating/eating disorders) and behavioural (type of sports participation) characteristics [8] but no comprehensive list is available and no single symptom(s) contribute to the diagnosis of the condition. Moreover, risk and case decisions may be difficult to determine because the links between LEA and the physiological abnormalities that characterise RED-S have not been established as causal. Rather they rely on cross-sectional and observational data often in athletes in sports which emphasise leanness or low-body weight, or athletes and other populations with eating disorders [11].

Measurement of risk factors for LEA and RED-S is also problematic. There is no standardised protocol for assessment or guidelines for calculation of energy availability (EA) [2, 12, 13]. To measure EA accurately, fat free mass (FFM), DEI and EEE are needed, all of which are labour intensive and prone to error. In females, an optimal EA of 45 kcal/ kg FFM/ day allows for healthy physiological functions with body systems becoming substantially perturbed at an EA of 30 kcal/kg FFM/day [5]. In males, the corresponding EA cut-points remain unclear [5]. To assess RED-S risk in athletes using the RED-S CAT, gold standard methods of measurement can be applied to the risk factors [8]. For example, measurement of bone mineral density and percentage of body fat using a dual-energy X-ray absorptiometry (DXA) [8] . However, such measures are often impractical for extensive application. Thus, accessibility, resource constraints, and athlete compliance with measures impede LEA/ RED-S screening. Also, the mismatch between when disordered eating behaviours and/or high EEE occurred and the assessed DEI and EEE means that measures are valid only at the point of assessment. Finally, the determination of LEA may not coincide with RED-S symptoms. For example, in female athletes menstrual disruption is not linked with any threshold value of LEA [14].

The challenges outlined in RED-S and LEA measurements mean that questionnaires may be frequently used for risk screening in athletic population. These questionnaires typically focus on disordered eating/ eating disorders. However, whilst LEA is more prevalent in athletic than sedentary populations, behaviours characterising LEA in sedentary individuals may not translate to pathological features in athletes. High EEE, and low body fat are also characteristics of athletic success in many sports [15]. The sensitivity of available questionnaires to distinguish athletes with or without LEA is debatable. Moreover, whether questionnaires objectively determine health and performance outcomes of LEA/ RED-S for different sexes is also arguable. Current questionnaires, such as Low Energy Availability in Females Questionnaire (LEAF-Q) [16], validated for female endurance athletes, and Sport-specific Energy Availability Questionnaire and Clinical Interview (SEAQ-I) [17], developed for male competitive road cyclists, isolate athletes of specific sexes or sport. As RED-S can affect many levels of athletes, it is crucial to be able to identify LEA risk factors and the presence of LEA/ RED-S associated symptoms early [18].

If questionnaires can protect athlete health and performance, they must be well-validated to screen for LEA/RED-S risk despite the challenges associated with assessment. Given the uncertainties surrounding the sensitivity of questionnaires to detect symptoms associated with LEA, this review aims to describe and critique available questionnaires as markers of LEA/ RED-S risk in athletes.

## Methods

This review aims to examine and critique the suitability of questionnaires that have been developed or used in previous studies to identify LEA/ Triad/ RED-S risk, in athletic populations, in the last 10 years. This duration was chosen to reflect recent updates in consensus statements in relation to the Triad [19], and the introduction of the term RED-S [4], along with much stronger recognition that EA and not energy balance is the underlying driving factor behind these conditions [3]. We thus wanted to examine tools in current use which are those within the selected time frame. A systematic search was conducted using the PubMed database, in accordance to the Preferred Reporting Items for Systematic Reviews and Meta-Analyses (PRISMA) guidelines. The key search terms included were: (Surveys and Questionnaires[meSH] OR (‘questionnaire’ OR ‘survey’)) AND (‘Relative Energy Deficiency in Sport’[meSH] OR ‘energy deficiency’ OR ‘Low energy availability’ OR ‘female athlete triad’ OR ‘triad’) AND (‘athletes’ OR ‘exercising men’ OR ‘exercising women’). Articles published between January 2010 and July 2020 were considered if they were published in English, and available in full text. The inclusion criteria were as follows:

1. If a questionnaire(s) was used in the study to identify LEA and/ or Triad and/or RED-S risk;
2. Studies that developed questionnaires to identify LEA and/ or Triad and/or RED-S risk;
3. Study participants belonged to an athletic population (athletes, recreational exercisers, dancers, etc);

In addition to the systematic search, additional papers were also identified through cross-checking of sources, and included for review. Duplicate articles were removed, and abstracts were screened for relevance. All articles included were assessed and agreed on by the two authors for suitability.

## Results

**Fig. 2:**
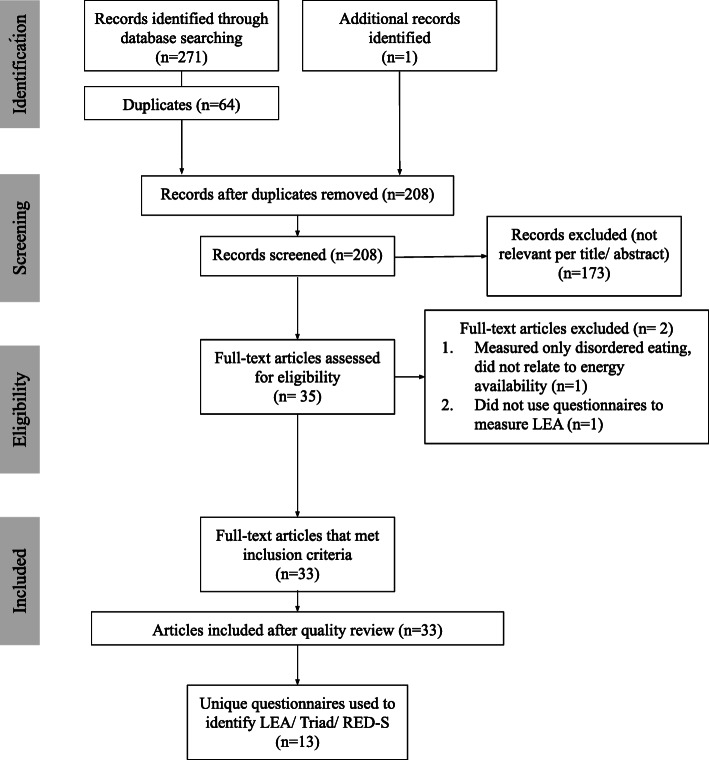
Search strategy, study selection process, and questionnaires selected

A total of 271 articles were found through the database search with one other article included after cross-checking. There were 64 duplicates removed and 175 articles that were not relevant after review of the abstract or full-text article (Fig. 2).

Based on the inclusion criteria, 33 articles were included for the review. 13 unique questionnaires were identified (Table 1). The questionnaires identified were categorised into three types: (i) measured LEA symptoms (*n* = 2); (ii) assessed proxy measures of LEA risk factors (*n* = 6); or (iii) measured LEA risk factors and symptoms (*n* = 5). Only three questionnaires included items related to EEE. Eight questionnaires had been validated for sensitivity, specificity, content validity, and test re-test reliability. Six questionnaires were designed specifically for females, one for males, and six were for use in both sexes.

**Table 1:** Questionnaires used in the assessment of Low Energy Availability (LEA) and Relative Energy Deficiency in Sport (RED-S)

Questionnaires	Validated in population	No. of items	Cut-off scores	Used as surrogate markers for	Validity and Reliability
Brief Eating Disorder in Athletes Questionnaire (BEDA-Q) [20]	Adolescent female elite athletes	9	An overall weighted score ≥ 0.27 indicates eating disorder [20]	Risk factors of LEA • Eating disorder screening ° Eating behaviours ° Weight concern ° Shape concern	Validated against EDI-2 Sensitivity: 82.1% (95% CI, 76.6–87.6) Specificity: 84.6% (95% CI, 79.4–89.8) Cronbach α: 0.8 1[20]
Eating Disorder Examination Questionnaire (EDE-Q) [21]	Non-active males and females	28	Dietary restraint score ≥ 3 and presence of ≥1 pathologic behaviour indicated LEA [22]	Risk factors of LEA • Eating disorder screening ° Shape, weight, eating concern and dietary restraint ° Disordered eating behaviours ▪ Binge-eating, lost control of eating, overeating, vomiting, laxatives usage, compulsive exercise	Sensitivity: 83% Specificity: 96% Positive predictive value: 56% [23] Cronbach α: 0.93 [24] Test-retest reliability Spearman’s rho > 0.86 [25]
Eating Disorder Inventory (EDI) – Drive for Thinness (DT) score [26]	Females	7	≥7 considered high [26]	Risk factors of LEA • Eating disorder screening ° Excessive concern with dieting, preoccupation with weight and fear of weight gain	Sensitivity: 86% Specificity: 80% [27] Cronbach α: > 0.80 [28] Test-retest reliability: 0.75–0.94 [29]
Eating Disorder Screening for Primary Care (ESP) [20]	Primary care patients for eating disorders and university students	4	≥3 in abnormal responses indicated LEA [30]	Risk factors of LEA • Eating disorder screening ° Eating behaviours ° Weight concern ° Family & self-report history of eating disorder	Sensitivity: 100% (95% CI, 90–100%) Specificity: 71% (95% CI, 0.0–0.15) [20]
Female Athlete Triad Risk Scale [31]	Not validated	6	≥3 indicated risk of Triad [31]	Risk factors and symptoms of LEA • Triad risk screening ° Eating behaviours ° Menstrual function ° Bone injury history	–
Female Athlete Triad Screening Questionnaire [32]	Not validated	12	Any positive answer to any questions indicated need for further measurements	Risk of factors and symptoms of LEA • Screening for Triad risk ° Disordered eating/ eating disorders ° Body image questions ° Menstrual history ° Bone Health	–
Low Energy Availability in Females Questionnaire (LEAF-Q) [16]	Female endurance athletes	25	≥8 indicated LEA [16]	Symptoms of LEA • LEA risk screening ° Menstrual function ° Injury ° Illness frequency ° Gastrointestinal function	Sensitivity: 78% Specificity: 90% Test re-test reliability: 0.79 Cronbach α: ≥ 0.71 [16]
Meal attitudes and body weight questions [33]	Not validated	2	Indicated to be at LEA when responses are: - Frequently lose weight intentionally - Consume less than 2 meals a day [33]	Risk factors of LEA • Screening for Triad risk ° Frequency of meals per day ° Body weight	–
RED-S risk measurement for cyclists [34]	Not validated	3	Indicated to be at LEA when ≥1 response on: - > 5% of body weight loss in the last month - > 14 days of missed training or competition because of illness, - > 20 missed days of training or competition because of injury [34]	Symptoms of LEA • Screening for RED-S risk ° Loss of body mass ° Injury and illness history	–
RED-S Specific Screening Tool (RST) (female and male versions) [35]	Female version: Middle and high-school female soccer players Male version: Not validated	25–31	Risk of RED-S Females < 16 years old/ non-menstruating and males (all ages): - Low < 100 - Moderate 101–400 - High > 400 Females > 16 years old - Low < 150 - Moderate < 150–500 - High > 500 [35]	Risk factors and symptoms of LEA • Screening for RED-S risk ° Menstrual function ° Activity levels ° Nutrition and diet ° Injury ° Physiological effects ° Psychological effects ° Factors that affect bone mineral density	Female version: Validated against Pre-Participation Gynaecological Examination Survey (r = 0.697, *p* < 0.001)
Sport-specific Energy Availability Questionnaire and Interview (SEAQ-I) [17]	Male road cyclists	6	–	Risk factors and symptoms of LEA • Screening for LEA risk ° Weight change ° Nutrition change ° Fuelling around training (e.g. weekly fasted rides) ° Bone injury history ° Illness history ° Medication history	Content validity measured by clinical sports endocrinologist, a sports research scientist, a registered sports performance dietician, competitive male cyclists and coaches
Three-Factor Eating Questionnaire (TFEQ) – Dietary cognitive restraint [36]	Non-obese and obese males and females	51	≥14 is considered as elevated	Risk factors of LEA • Dietary restraint	Sensitivity: 72% Specificity: 70.1% [37] Internal consistency: 0.93 [36] Cronbach α: 0.71 [37]
Triad consensus panel screening questions by the Female Athlete Coalition [19]	Not validated	11	–	Risk factors and symptoms of LEA • Screening for Triad risk ° Menstrual function ° Weight concern ° Eating behaviours ° Eating disorder history ° Bone function	–

### Brief eating disorder in athletes questionnaire (BEDA-Q)

The BEDA-Q distinguishes adolescent female elite athletes with and without eating disorders by using a weighted equation score based on nine questions – an athlete who scores ≥0.27 is classified to be at risk of an eating disorder [20]. Robertson and Mountjoy [38] recommended the combination of BEDA-Q and LEAF-Q for RED-S screening in female artistic swimmers to gain greater insights on eating behaviours that may cause intentional LEA. Ackerman et al. [30] used BEDA-Q as a surrogate marker for LEA in 1000 female athletes (15–30 years old) – 39.1% (*n* = 391) were identified as having LEA. Although the BEDA-Q is only validated in female elite adolescent athletes, the items are not female-specific and potential exists for use in male athletes.

### Eating disorder examination questionnaire (EDE-Q)

The EDE-Q [21] measures disordered eating psychopathology. It includes four subscales – Dietary Restraint, Shape Concern, Eating Concern, and Weight Concern, and a global score (average of subscales). Frequency of six disordered eating behaviours (e.g. binge eating, compulsive exercise) were also assessed [39]. Compulsive exercise is measured by the item – “how many times have you exercised in a “driven” or “compulsive” way as a means of controlling your weight, shape or amount of fat or to burn off calories?”. The EDE-Q has been used as a potential screening tool for Triad risk [40], and can also identify males with eating disorder symptoms [41]. The EDE-Q covers risk factors including behaviours associated with DEI and EEE – tendencies that lead towards intentional LEA.

The EDE-Q has been used in adults and adolescents of both sexes [42, 43], in athletic populations and elite para athletes [22, 44]. It was used as a surrogate marker for LEA, and measured Triad components in female athletic populations [45, 46]. Moreover, the EDE-Q (Dietary Restraint and six pathologic behaviours) determined LEA in elite para athletes [22]. A Dietary Restraint score of ≥3 and presence of ≥1 pathologic behaviour indicated LEA [22]. EDE-Q assesses compulsive exercise which can indicate LEA, a component of questioning often omitted from other screening tools assessing LEA risk [13]. One concern is that the clinical EDE-Q cut-off scores may differ across sexes. Males appear not to score as highly as females [41]. Hence, the cut-off scores must be selected with caution. Overall, the EDE-Q is a potentially suitable surrogate measure for LEA prevalence, or even RED-S risk in large male and female athletic populations, when biochemical parameters cannot be measured. However, because the measures on the EDE-Q are related to body image, weight concern and behaviours related to dietary restraint these may precede any symptoms associated with LEA or RED-S themselves.

### Eating disorder inventory (EDI) - drive for thinness (DT) score

The DT subscale is part of the Eating Disorder Inventory-2 (EDI-2) that measures eating disorders symptoms [26]. The DT score measures disordered eating attitudes associated with body image, weight, and shape, a score of ≥7 is considered high [26]. The DT subscale may be an appropriate proxy indicator for LEA, as strong relationships between DT score with physiological measures (e.g. suppressed thyroid activity) associated with LEA exist [47, 48]. Moreover, severe menstrual disturbances in exercising women have also been associated with high DT score [47, 48]. The DT score may reflect behavioural changes, such as energy restriction in pursuit of a thin ideal, which may result in LEA [47]. However, the EDI-DT subscale has not been used to identify LEA in males. Hence, the appropriateness of DT as a surrogate marker for LEA should be further studied. Notably, DT items are not sex-specific and have been used in male populations (unrelated to LEA) [49].

### Eating disorder screen for primary care (ESP)

The ESP originally screened for eating disorders in primary care patients and university students [50], but has since been applied in athletic populations. The ESP was developed from previous studies [51–53] and validated against the SCOFF (Sick, Control, One stone, Fat, Food) clinical prediction questions [54], using the Questionnaire for Eating Disorders Diagnosis [55] as the independent standard. The ESP showed to be more sensitive than SCOFF, and was hence deemed useful for eating disorders screening.

Ackerman et al. [30] used ESP as a surrogate marker for LEA in female athletes. 12.3% (*n* = 123) were identified to be at risk of LEA, as indicated by score: ≥3 in abnormal responses. This was considerably lower than the 39.1% diagnosed by the BEDA-Q used in the same study. Ackerman and colleagues suggested that the BEDA-Q may be more inclusive than the ESP [20]. They reflected that the questions in the ESP focus on eating behaviours (satisfaction with eating patterns and secret eating), feelings related to body weight, and whether there have been past diagnoses of eating disorder for the individual or their immediate family, whereas the BEDA-Q includes additional questions about body image and perfectionism. The limited assessment areas within the ESP may restrict its ability to diagnose LEA in athletes where body composition targets often go beyond simple relationships with food or weight and where there is a drive toward leanness [18]. Thus, the ESP alone may best be used with other tools to improve the overall ability to detect LEA rather than as a sole surrogate marker of LEA. Finally, while the ESP has yet to identify LEA in male athletes, it is not sex-specific and has previously been used to screen for eating disorders in male and female student athletes [56].

### Female athlete triad risk scale

The Female Athlete Triad Risk Scale [31] assessed Triad risk in young adolescent and adult female figure skaters, dancers, and runners (*n* = 712). The items were adapted from the Preparticipation Screening for the Female Athlete tool [57], Female Athlete Screening Tool [53] and LEAF-Q [16]. In this study, 60% of female athletes were considered at risk of the Triad as they endorsed ≥3 of the six questions [31]. This tool could potentially measure Triad risk in large numbers of female athletes due to the small number of items and clear cut-off point. However, the validity and reliability of the tool has yet to be tested and the items are female-specific.

### Female athlete triad screening questionnaire

The Female Athlete Triad Screening Questionnaire, addresses all Triad components [32], and measures Triad risk in athletes prior to the competitive season. This questionnaire consists of yes/no responses, which makes it convenient. There is no stated cut-off score for this tool, however it has been stated that a positive response to any items indicates the need for clinical in-depth evaluation [32]. However, the questionnaire has limited application in male athletes as it contains female-specific items. While this tool is designed primarily for female athletes, only one item is sports specific (Do you try to lose weight to meet weight or image/appearance requirements for your sport?). The lack of established validity and reliability makes it unclear if it is specific and sensitive enough to be used in further studies. Additionally, the aspect of EEE remains unaddressed.

This questionnaire measured Triad risk in triathletes, where 24% (*n* = 75) indicated at least one Triad component, and 8% (*n* = 25) all components [58]. Another study used part of this questionnaire (presence of eating disorder, dietary habits, worrying about weight/ body image) to measure the energy-deficiency component of the Triad in female collegiate rowers [59]. The energy deficiency related components did not differ between lightweight and openweight rowers, albeit the study hypothesised that lightweight rowers appear to be at higher risk of the Triad.

### Low energy availability in female questionnaire (LEAF-Q)

The LEAF-Q detects female endurance athletes at risk of LEA by examining self-reported LEA associated physiological symptoms which includes gastrointestinal and menstrual function (Table 1). A score of ≥8 out of 49 indicates risk in female endurance athletes [16].

The LEAF-Q commonly measures LEA risk in large, exercising cohorts. It was found that almost 40% (*n* = 331) of active females in Ireland were at-risk of LEA [44]. The LEAF-Q can also be used with disordered eating screening tools. Folscher et al. [60] used the LEAF-Q and Female Athlete Screening Tool [53] to determine the Triad risk (44.1%, *n* = 135) in ultra-marathon female athletes. One-third of at-risk participants showed disordered eating behaviours (e.g. restrictive eating), as indicated by the Female Athlete Screening Tool. The combination of questionnaires can provide deeper understanding of the cause of LEA.

A criticism of LEAF-Q is its application in female athletes only. As nearly half the items (*n* = 12) relate to menstrual function, the current cut-score (≥8) [16], would underestimate male athletes at LEA risk. Slater (2015) (unpublished observations) proposed using the calculated average scores of the non-menstrual questions for females classified as LEA risk, as an alternative cut-off point score for males to allow better risk comparison between sexes. However, this requires further validation, as menstrual dysfunction is a core feature of LEA in women and there is no acceptable substitute feature for men in the modified questionnaire. Moreover, it is uncertain if other physiological symptoms measured in the LEAF-Q, would affect males with LEA comparatively to the same degree as females. Furthermore, LEAF-Q does not consider EEE.

### Meal attitudes and body weight questions

Two questions were used to determine LEA risk in female Japanese collegiate athletes (*n* = 531] [33]. LEA risk was identified when participants answered that they usually consume meals less than twice per day, and frequently lose weight intentionally – 2.7% athletes were identified to be at risk [33]. The advantage of the approach is that the two items are easy to understand and answer. Moreover, they are not sex-specific and potentially could be applied in both sexes. However, the validity and reliability of the items have not been tested or used in further studies. Hence, it is unclear they are specific and sensitive enough to provide a comprehensive assessment of LEA risk. No questions related to exercise were included.

### RED-S specific screening tool (RST)

The RST (male and female version) was developed for Triad and RED-S screening in young athletes [35]. The RST contains components from Pre-Participation Gynaecological Examination (PPGE) [61] and Eating Disorders Screening Tool [62] . The female version of the RST was validated in female adolescent soccer players (*n* = 39), against the PPGE (*r* = 0.697, *p* < 0.001). The scoring determines the risk level for RED-S (low – moderate – high). There are specific cut-off points for males, and females after or before the onset of menarche and/or older or younger than the age of 16 years.

This tool has several advantages over other questionnaires in this review. In addition to being designed for application to both sexes and across ages, it considers several risk factors and symptoms of RED-S/ LEA (Table 1). The RST accounts for activity levels (i.e. how many hours of physical activity do you do every day). More hours spent on physical activity constitutes a higher risk of LEA. Furthermore, the RST is applicable for a multidisciplinary team to administer.

The RST is relatively new and has addressed a major gap in the literature – a RED-S/ LEA screening tool applicable to male athletes. However, a more robust validation may be necessary for the female version (i.e. validating it against biomarkers of RED-S/ LEA). Moreover, although a version has been designed for male athletes it is presently not validated. Thus, it remains unclear if it is sensitive and specific enough to address RED-S risk in male athletes. Nevertheless, the RST represents a potentially suitable surrogate marker to measure RED-S risk in large populations of male and female adolescent and possibly adult athletes, when biochemical parameters cannot be measured.

### RED-S risk measurement for young male cyclists

This questionnaire assesses RED-S risk in young male cyclists (17–23 years old) [34]. RED-S risk was considered elevated, if ≥1 of the following answers were recorded – more than 5% of body weight loss in the last month, at least 14 days of missed training or competition because of illness, or at least 20 missed days of training or competition because of injury. In the study, 44.6% (*n* = 21) cyclists had an elevated risk for RED-S and there was a negative association between risk level with performance determined as relative peak power. The items in the questionnaire are straightforward, and are neither sex nor sports specific, there is potential for wider use in female athletes and other sports. However, this questionnaire has not been validated, and makes no measure of EEE preferring to focus on symptoms associated with overtraining.

### Sport-specific questionnaire and clinical interview (SEAQ-I)

The SEAQ-I identifies male cyclists at risk of RED-S and categorises EA on a scale from adequate, to acute intermittent, to chronic. Apart from content validity, this tool has not been validated further. The reliability of this tool is not tested. Thus, while the SEAQ-I attempts to fill a large gap in the current literature – the lack of male-specific questionnaires that address LEA/ RED-S risk - it lacks validity and reliability. It may be difficult to apply this tool to further studies as the questions are brief and related to cycling history and nutrition.

### Three-factor eating questionnaire (TFEQ)

The TFEQ contains 51 items that measures human eating behaviour: (1) dietary cognitive restraint, (2) disinhibition, and (3) hunger [36]. Only the dietary cognitive restraint subscale has been used in athletes [63], as it is related to LEA and consists of items related to weight control. A score of ≥14 is considered as elevated [36]. However, this score may underestimate the risk of LEA in athletes [47]. Moreover, a modified score of 9 has been previously used, to suggest elevated dietary cognitive restraint as a risk factor associated with LEA and Triad risk in athletes [63].

The DT subscale was found to be positively correlated with dietary cognitive restraint (*r* = 0.602, *p* < 0.001), indicating that these two measures are tightly coupled [47]. Both measures indicate a stable disposition to limit food intake and hence a likelihood of LEA [36]. However, it has been mentioned that the dietary cognitive restraint subscale was unsuccessful in discriminating energy deficient women from energy balanced women (when biomarkers were measured) [47]; hence it is unclear if DT is an appropriate LEA marker and further studies should investigate this in both sexes. Furthermore, TFEQ does not consider EEE.

### Triad consensus panel screening questions by the female athlete coalition

The Triad consensus panel screening questions by the Female Athlete Coalition are incorporated in the Triad Cumulative Risk Assessment Tool [19]. This questionnaire serves as a pre-screening tool and indicates the need for an in-depth evaluation for Triad [19]. However, there is no clear cut-off point for the requirement of further evaluation hence this questionnaire cannot be used independently without follow-up physiological assessments. Furthermore, this tool has to be validated and EEE is also not measured.

Five of the 11 items within the questionnaire are female-specific hence limiting its application for use for men. Nevertheless, a previous study used the Cumulative Risk Assessment Tool [19] to assess LEA in male and female elite distance athletes [64]. For male athletes, the tool was applied by replacing menstrual function (i.e. amenorrhoea) with low testosterone scores. However, testosterone was measured in blood samples and the difficulties of assessing testosterone levels without a clinical measure suggest that this adaptation has little value in large populations. Hence, even though the tool can be adapted for both sexes, the screening questionnaire does not provide value for male athletes without follow-up measurements.

## Discussion

This review identified thirteen questionnaires that have assessed the prevalence of LEA, RED-S or the Triad in research studies over the past decade, used across a broad spectrum of athletes (recreational, competitive, elite), and in different types of sport (i.e. endurance running, gymnastics). It is notable that in many cases the use of these questionnaires in these various athletic populations was not that of the intended population that the questionnaire was developed for or validated in.

It is crucial to note that questionnaires are not designed to be the definitive measure in identifying or diagnosing LEA and should not be used as the sole screening tool, but instead as a primary screening tool for identification of those at risk in field settings. When questionnaire responses indicate an elevated risk, a thorough health screening to address any implications is warranted, and the decision on further sports participation should be made with a multidisciplinary support team (physician, dietitian, exercise physiologist, psychotherapist). The questionnaires can be used with physical or physiological measures and clinical assessments to support any diagnosis. Nevertheless, using questionnaires to estimate LEA risk has the advantages of convenience, speed of assessment, cost-effectiveness for large-scale screening of athletes, and epidemiological research.

### Frequently used validated questionnaires

The most frequently used validated questionnaires for determination of LEA is the LEAF-Q (16 out of 33 studies) and EDE-Q (4 out of 33 studies). LEAF-Q does not measure actual LEA behaviours – eating disorders, disordered eating or high EEE – but instead, symptoms related to LEA. Conversely, the EDE-Q is a behavioural questionnaire that focuses on eating behaviours, body satisfaction, and briefly on exercise behaviour. The EDE-Q, however, was not designed to measure LEA in athletes despite widespread application within this group. Nevertheless, the EDE-Q can be applied to both sexes. As no Low Energy Availability in Males Questionnaire is yet available [5], the EDE-Q may be the preferred measure when comparing LEA risk between sexes. Although it contains only 28 items, some researchers only applied the DR subscale and pathological behaviours assessment when time constraints could be an issue. These two aspects can be directly associated with DEI and EEE [22]. Given that the LEAF-Q and EDE-Q measure symptoms and behaviours, respectively, there is potential to apply these two questionnaires in a complementary manner but whether this improves diagnosis of LEA has yet to be shown. It is important to note that there was no single gold standard for validation of each questionnaire and not all questionnaires had the same validation process (as shown in Table 1). Hence it can be difficult to compare the validity of one questionnaire to another. Also, apart from the LEAF-Q, there were no other questionnaires that were specifically validated with measurements related to LEA consequences (e.g. validating items related to bone health with bone density measured by DXA even though the questionnaires (e.g. EDE-Q) have been used to indicate LEA prevalence in previous studies [65]. This further emphasises the need for validation of questionnaires that measure LEA risks against gold standard methods of assessing the consequences of LEA.

### Questionnaires for male athletes

Male athletes seem to be at lower risk of developing eating disorders/ disordered eating than their female counterparts [56]. Nevertheless, a high prevalence of these disorders has been found in male athletes involved in cycling, gravitational sports and weight class sports [66]. However, attempts to develop specific questionnaires to assess risk of LEA/ RED-S in male athletes are recent and limited.Three questionnaires in this review were developed specifically for male athletes, of which two were for cyclists – the SEAQ-I and RED-S risk measurements for young male cyclists. Application of the same or adapted versions of these questionnaires in other sports has yet to be shown. Nevertheless, among the existing pool of questionnaires, there are others that do not contain female-specific items which can potentially be used in males –ESP, EDI-DT, BEDA-Q, EDE-Q, Questions on meal attitudes and body weight, and TFEQ. Better validation of all these questionnaires to address LEA in male athletes is needed to ensure accuracy of the screening process.

Male athletes can experience LEA and the consequences are similar to those in female athletes [4, 67]. However, there are no validated symptom-based questionnaires similar to the LEAF-Q applicable in men. Better characterisation of the presence and severity of symptoms/ abnormalities associated with LEA and RED-S, and how they differ in extent to their female counterparts, is warranted for male athletes [5, 17, 66]. The overall void of validated questionnaires suitable for male athletes limits large population-based studies and understanding of the prevalence and impact of LEA in men remains a concern.

### Measurement of LEA risk factors vs measurement of symptoms/ outcomes

LEA risk has been indirectly measured through questionnaires that measure behavioural risk factors or LEA symptoms. Some questionnaires reviewed screen for disordered eating/ eating disorders (BEDA-Q, EDE-Q, RST, TFEQ, EDI, ESP) and were developed some decades ago. In most instances they have not been revised to include the most recent Diagnostic and Statistical Manual of Mental Disorders (DSM-5) criteria, except for the BEDA-Q. Hence, future studies that intend to adopt these questionnaires as surrogate markers for LEA must do so with caution, according to the research aims. Moreover, while these questionnaires account for the risk of disordered eating/ eating disorders, high EEE as risk factors for LEA are not assessed using these tools (except EDE-Q and RST).

Excessive exercise has been associated with an increased risk of LEA [2, 68]. A positive association between exercise dependence scores and disordered eating symptoms has been established; this includes for individuals who do not increase their DEI with higher EEE, which can lead to pronounced LEA [68]. Excessive exercise or exercise addiction can be measured through questionnaires such as the Exercise Addiction Inventory (EAI) [69], Compulsive Exercise Test [70] and the Exercise Dependence Questionnaire [71]. Nevertheless, measurement of exercise behaviours was limited within this review. Researchers must realise the importance of measuring exercise behaviours when assessing for LEA, Triad or RED-S. As LEA may be the inadvertent failure to increase energy intake when undertaking high exercise volumes. Thus, LEA can occur without the presence of disordered eating behaviours/ eating disorders, or even mismanaged efforts to reduce body size or body composition [3]. As the extent to which each cause contributes to LEA is uncertain, we emphasise here that researchers and support teams who screen for LEA need to carefully chose a tool, or a combination of tools, which can account for all possible origins of the LEA (dietary, behavioural, and exercise).

In a practical setting, a combination of questionnaires covering the various dimensions of LEA (symptoms and behavioural risk factors) can be applied anually for screening, or in periods of heavy training and competition, in male and female athletes. It is crucial to identify and apply these questionnaires to higher risk groups of athletes, such as those with poor nutritional knowledge or those involved in leanness demanding sports. Nevertheless, the validity of this approach needs to be tested to determine whether questionnaire fatigue exists and whether the sensitivity of diagnosis is improved.

### Limitations and prevailing gaps

Questionnaires are useful tools in early detection of LEA risk. However, not all questionnaires used in published studies have been validated in athlete specific populations. Eight out of 13 questionnaires have been validated but only half in an active population (BEDA-Q, LEAF-Q, SEAQ-I, RST), while the other half seemingly remain unvalidated (EDE-Q, EDI, ESP, TFEQ). Furthermore, due to the self-report nature of questionnaires, response bias and under-reporting may exist. Hence, responses must be interpreted with caution and other forms of athlete monitoring should be used where possible. Moreover, validity, reliability, and measurement error were not provided for all questionnaires reviewed (Female Athlete Triad Risk Scale, Female Athlete Triad Screening Questionnaire, Meals attitudes and body weight questions, RST, Triad consensus panel screening questions).

Furthermore, this narrative review is limited to papers from 2010 to 2020, the English language, and also one database. There are still prevailing research gaps – more questionnaires are needed to address i) exercise and physical activity levels; ii) LEA items specific to male athletes; iii) items that extend beyond the Triad to assess other outcomes of RED-S.

## Conclusions

This review provides novel insights on the questionnaires currently used to monitor or measure LEA risk in athletes. The questionnaires identified can act as surrogate markers to estimate LEA risk in large populations, when resources are not readily available or in field settings. As RED-S can impair athletes’ health and performance, these questionnaires can help indicate any disordered eating behaviour or excessive exercising patterns early. However, while they can identify athletes with intentional energy restriction they are limited in their effectiveness to identify athletes who fail to increase energy intake with increased training demands. Importantly, questionnaires should only be regarded as screening measures and not diagnostic tools for LEA, RED-S or the Triad. In-depth follow-up which should include physiological measurements is necessary with a qualified support team if there are any indications of LEA risk to prevent escalation of the condition.

## Data Availability

Data sharing is not applicable to this article as no datasets were generated or analysed during the current study.

## Abbreviations

BEDA-Q: Brief Eating Disorder in Athletes Questionnaire
DEI: Dietary Energy Intake
DT: Drive for Thinness
DXA: Dual-Energy X-Ray Absorptiometry
EDE-Q: Eating Disorder Examination Questionnaire
EDI: Eating Disorder Inventory
EEE: Exericse Energy Expenditure
ESP: Eating Disorder Screen for Primary Care
IOC: International Olympic Committee
LEAF-Q: Low Energy Availability in Female Questionnaire
RED-S CAT: RED-S Clinical Assessment Tool
RED-S: Relative Energy Deficiency in Sport
RST: RED-S Specific Screening Tool
SEAQ-I: Sport-specific Energy Availability Questionnaire and Clinical Interview
TFEQ: Three-Factor Eating Questionnaire
Triad: Female Athlete Triad
